# Whole Span Plating Prevents Subsequent Ipsilateral Femoral Fractures After Peri-Implant Fixation: A Preliminary Report

**DOI:** 10.3390/jcm14103473

**Published:** 2025-05-15

**Authors:** Shunsuke Sato, Satoshi Hatashita, Michiyuki Hakozaki, Takuya Kameda, Yoichi Kaneuchi, Masayuki Ito, Yoshihiro Matsumoto

**Affiliations:** 1 Department of Orthopaedic Surgery, Fukushima Medical University School of Medicine, Fukushima 960-1247, Japan; paco@fmu.ac.jp (M.H.); a0001826@fmu.ac.jp (T.K.); kaneuchi@fmu.ac.jp (Y.K.); ymatsu@fmu.ac.jp (Y.M.); 2 Department of Traumatology and Reconstructive Surgery, Fukushima Medical University School of Medicine, Fukushima 960-1247, Japan; hatashi@fmu.ac.jp (S.H.); aitrsc@fmu.ac.jp (M.I.); 3 Traumatology and Reconstructive Surgery Center, Aizu Chuo Hospital, Fukushima 965-0011, Japan

**Keywords:** peri-implant fracture, periprosthetic fracture, whole span plating, osteoporotic fracture, fragility fracture

## Abstract

**Background:** Peri-implant femoral fractures are increasingly prevalent among the elderly, often leading to re-fractures due to osteoporosis and implant stress. Whole span plating (WSP) has been proposed as a surgical approach to mitigate this risk. **Methods:** A retrospective study was conducted on 10 patients (mean age: 79.5 years) who underwent WSP for peri-implant femoral fractures between April 2020 and March 2023. Fractures were classified using the Vancouver, NPPIF, and Lewis and Rorabeck systems. Surgical indication required meeting at least two of the following criteria: age ≥ 70 years, history of fragility fracture(s), high fall risk, severe osteoporosis, extensive fracture pattern, or no implant loosening. **Results:** No re-fractures were observed during a mean follow-up period of 14.5 months. Although 90% of patients required red blood cell transfusions (mean volume: 3.8 units), early weight-bearing was achieved in most cases. Four patients had received osteoporosis treatment, highlighting the need for integrated bone health management. **Conclusions:** WSP appears to be a feasible and safe surgical option for high-risk patients with peri-implant femoral fractures, potentially reducing the incidence of subsequent fractures. Further studies with larger cohorts and longer follow-up are warranted.

## 1. Introduction

**Figure 1:**
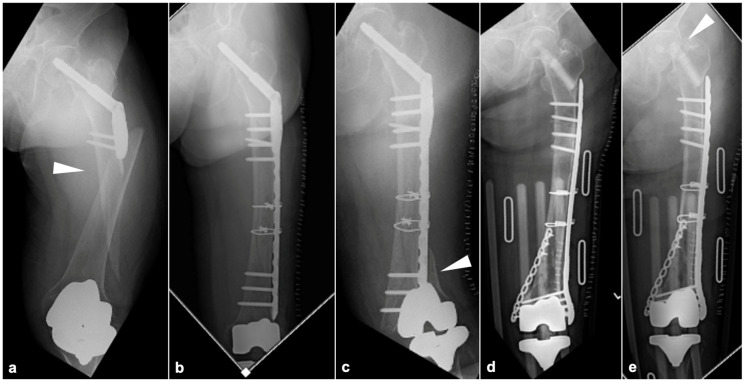
Images of specific cases illustrating the ‘fracture cascade’. (**a**) A compression hip screw (CHS) was used for this proximal femur fracture, resulting in a new fracture distal to the plate (white arrowhead). (**b**) The fracture shown in panel A was internally fixed using a locking compression plate on the lateral side of the femur. (**c**) After the condition presented in (panel **b**), a new fracture occurred between the distal part of the plate and the femoral component of the TKA (white arrowhead). (**d**) After the removal of the CHS, internal fixation of the distal femur fracture was performed with a plate from the medial and lateral sides. (**e**) A new femoral neck fracture occurred after the situation presented in (panel **d**) (white arrowhead).

Populations worldwide are aging and healthy life expectancy is increasing [1]. The number of older adults undergoing an arthroplasty in order to preserve their activities of daily living (ADLs) is thus also increasing [2]. However, the number of patients with periprosthetic fractures is increasing because of the higher risk of fractures as bones become more fragile with age [3,4,5]. The use of internal fixation for proximal, femoral shaft, and distal femoral fractures is increasing [6], and non-prosthetic peri-implant fractures (NPPIFs) are a potential complication of the application of internal fixation for such fractures [7]. Even after the internal fixation of such peri-implant femoral fractures, re-fractures can occur outside the internal fixation material, resulting in the ‘fracture cascade’ (Figure 1) [8].

Osteoporosis is a major risk factor for fragility fractures, including periprosthetic and peri-implant fractures. Although fracture risk is correlated with bone mineral density (BMD), BMD alone does not fully explain fracture susceptibility [9]. Recent clinical guidelines emphasize the importance of using comprehensive tools such as the Fracture Risk Assessment Tool (FRAX) to evaluate overall fracture risk and initiate appropriate pharmacologic treatment, even in patients with “normal” bone density but a low-energy trauma history [9]. Notably, patients with a prior fragility fracture—especially of the ankle, foot, or hip—should be considered clinically osteoporotic and treated accordingly, as their risk of subsequent fracture is markedly elevated. Furthermore, osteoporosis is frequently underdiagnosed in elderly individuals undergoing arthroplasty, despite being a known risk factor for periprosthetic fractures [9]. These findings highlight the importance of proactive bone health evaluation and management in preventing fracture cascades.

**Figure 2:**
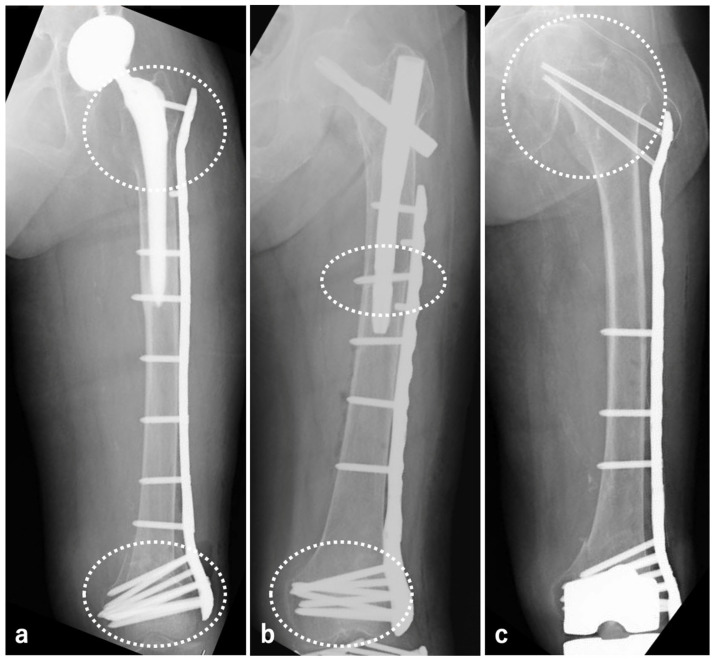
In peri-BHA/THA fractures (**a**), peri-SFN fractures (**b**), and peri-TKA fractures (**c**), the areas circled with white dots were covered with WSP to prevent re-fracture and to strengthen fixation.

To address this issue surgically, our institution employs the whole span plating (WSP) method in cases of peri-implant femoral fractures, covering the entire femur. Unlike previously reported surgical techniques referred to as WSP, our method enables fixation that includes areas of possible re-fracture (Figure 2). We believe this method may be particularly useful in elderly individuals with a history of fragility fractures or repeated falls, who represent a high-risk subgroup for subsequent ipsilateral femoral fractures. This present investigation was performed to determine the efficacy of WSP for preventing postoperative re-fracture in cases involving peri-implant femoral fractures.

## 2. Patients and Methods

This was a retrospective descriptive study of 10 cases of peri-implant femoral fractures treated with WSP at the Aizu Chuo Hospital Traumatology and Reconstructive Surgery Center over a 3-year period from April 2020 to March 2023. The analyzed parameters included patient age, sex, type of pre-existing implant, classification of the peri-implant fracture, history of fracture(s), comorbidity profile, preoperative osteoporosis treatment history and corresponding medication details, American Society of Anesthesiologists physical status (ASA-PS) classification, red blood cell (RBC) transfusion volume, intraoperative blood loss volume, operative time, duration of non-weight-bearing period, pre-injury activities of daily living (pre-Injury ADLs), ADLs at the final follow-up, length of hospital stay, postoperative complications, pre-admission living situation, discharge destination, follow-up period, and the presence or absence of re-fracture at the final follow-up.

The peri-implant fractures were classified using the Vancouver classification for the bipolar hip arthroplasty (BHA)/total hip arthroplasty (THA) cases [10], the NPPIF classification for the short femoral nail (SFN) cases [7], and the Lewis and Rorabeck classification for the total knee arthroplasty (TKA) cases [11]. The Vancouver classification system divides post-THA femoral fractures into the following three categories: Type A includes fractures of the greater or lesser trochanter. Type B includes fractures around the stem. Type C includes fractures distal to the stem. The NPPIF classification is used for periprosthetic fractures around non-arthroplasty implants. It categorizes fractures based on a combination of factors: the type of implant (Type P for a plate implant and Type N for a nail implant), the fracture location (Type 1 for fractures at the implant tip and Type 2 for fractures at a site distant from the implant), and the healing status of the original fracture (Type A for healed fractures, Type B for unhealed fractures, and Type C for delayed union or nonunion). The Lewis and Rorabeck classification is used for post-TKA supracondylar femoral fractures. It categorizes fractures into type 1, which includes non-displaced fractures; type 2, which includes displaced fractures; and type 3, which includes cases with femoral component loosening, regardless of the displacement status.

### 2.1. Surgical Indications

The indication criteria for WSP were patients who met at least two of the following criteria: (i) age ≥70 years, (ii) history of fragility fracture(s) at other site(s), (iii) high risk of falls due to a medical condition, (iv) severe osteoporosis defined by the World Health Organization (WHO) as a condition in which the patient’s bone mineral density (BMD) is decreased by >2.5 standard deviations (SD) below the young adult mean (YAM) (T-score ≤ −2.5), in addition to the presence of at least one fragility fracture, (v) extensive fracture extent, (vi) no loosening of the implant.

### 2.2. Preoperative Planning

**Figure 3:**
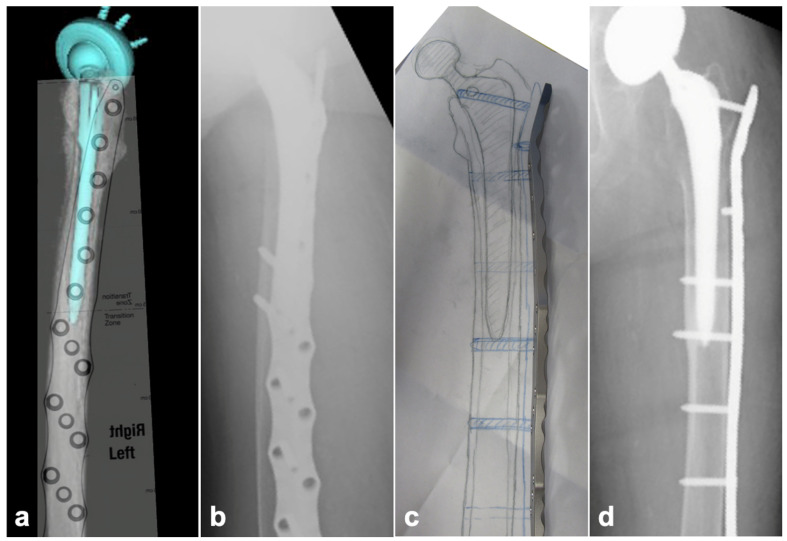
Preoperative planning for WSP. (**a**) Preoperative templating using 3D-CT. The position of the plate and the position and orientation of the screw insertion were determined in relation to the existing implant. (**b**) A postoperative lateral radiograph. Screws were inserted in the expected preoperative position. (**c**) A 100% sterilized drawing for use in the operative field. The operation could be started after the pre-bending of the implant of the expected size as per the drawing, thus reducing the operation time. (**d**) A postoperative anteroposterior radiograph. The implant was formed and placed almost exactly as drawn.

Surgery was planned based on the implant that had been inserted preoperatively in each patient. Preoperative planning involved using a three-dimensional computed tomography (3D-CT) image to visualize existing implants and bone morphology. A scaled plate template was overlaid on the CT images to determine optimal screw insertion points and trajectories (Figure 3a). To reduce operating time, full-scale sterile drawings were brought into the operating field and used as a reference to pre-bend the implant (using a plate bender) and shape it accordingly. (Figure 3b–d).

### 2.3. Surgical Technique

The surgical techniques used for WSP varied depending on the type of peri-implant fracture and are described below.

#### 2.3.1. BHA/THA

**Figure 4:**
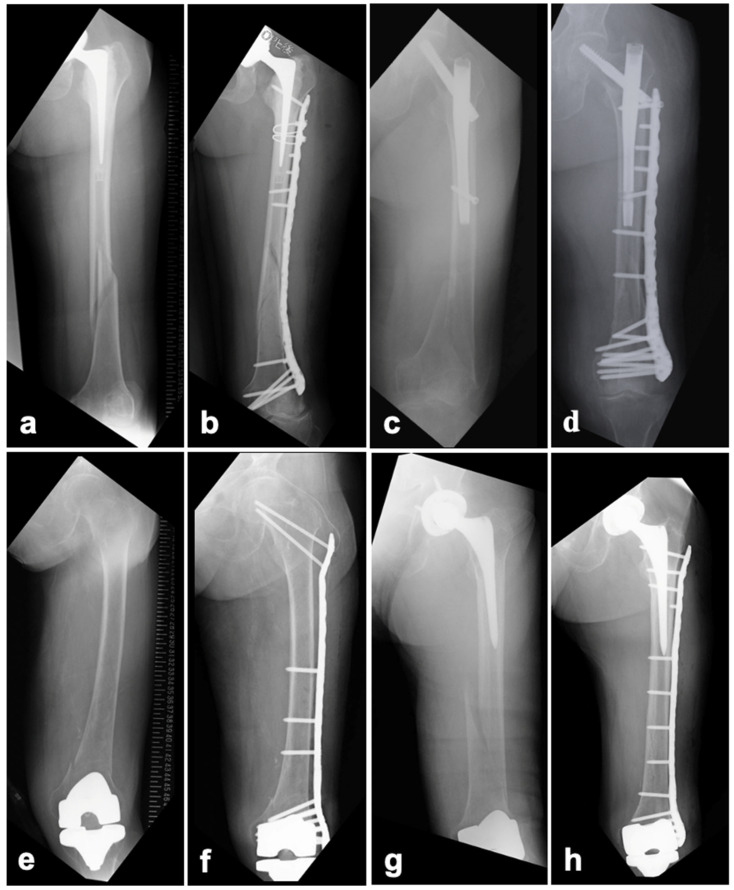
WSP in cases involving peri-BHA/THA, peri-SFN, peri-TKA, and interprosthetic femoral fractures. Preoperative (**a**,**c**,**e**,**g**) and postoperative (**b**,**d**,**f**,**h**) radiographic images.

The proximal side of the plate was pre-bent and placed in the greater trochanter region (vastus tubercle). The screws were inserted proximally from the plate to fix the trochanteric area and distally to insert locking screws as far as possible into the condylar area (Figure 4a,b).

#### 2.3.2. SFN

A long plate was placed without interference from the lag screw. The locations of the screw holes in the plate corresponding to the distal lateral screw holes in the SFN were planned during the preoperative drawing stage. The wire used for the provisional fixation of the plate was passed from the screw holes in the plate to the distal transverse screw holes in the SFN. In this plate position, the distal part of the plate was in a good position to be installed with the femoral condyle bone fragment, and the distal bone fragment was provisionally fixed to the plate. The screw diameter inserted from the plate was 5 mm and the screw had a 30° polyaxial function; therefore, the screw from the plate could be inserted into the distal transverse screw hole of the SFN, where a 5 mm dia. locking screw could also be inserted. This method has been reported as the “nail-plate docking technique [12]” including the rotational dislocation of the proximal bone fragment, and it was used in the present patient series. Distally, as in the BHA/THA cases, a locking screw was inserted as far as possible into the condyle (Figure 4c,d).

#### 2.3.3. TKA

Proximally, the plate was pre-bent as in a BHA and was placed up to the vastus tubercle. Proximal screws were inserted over the plate into the femoral neck and head. Locking screws were inserted as far as possible into the condyle without interfering with the femoral component (Figure 4e,f).

In all three methods, the screw insertion was performed using a minimally invasive solution targeting (MIS) device. The skin incisions were limited to those for plate insertion, fracture reduction, and where using the MIS device was not possible due to the pre-bend portion of the plate.

## 3. Results

The cases of 10 patients (two males, eight females) with a mean age of 79.5 years (range: 54–95 years) were analyzed (Table 1). The number of pre-existing implants varied among the patients. Six patients had BHA/THA stems. According to the Vancouver classification, one case was classified as Type B1, four as Type C, and one as Type B1+C. Two cases had an SFN for trochanteric fractures; both were classified as Type N2A based on the NPPIF classification, with one case involving a distal femoral shaft fracture and the other involving a supracondylar femoral fracture. One patient underwent a TKA and was classified as type 2 according to the Lewis and Rorabeck classification. An interprosthetic femoral fracture was involved in one patient’s case [13], in which both a THA and a TKA were performed on the same side classified as Vancouver Type C (Figure 4g,h).

A history of fractures at other sites before surgery was observed in five patients, three of whom had undergone multiple fracture treatments. Regarding medical comorbidities, six patients had a high risk of falls due to a history of cardiovascular or cerebrovascular disease, and five patients had severe osteoporosis (Table 1). The ASA-PS classification was Stage 2 in six cases and Stage 3 in four cases, and surgery was deemed feasible for all 10 patients. An NCB Periprosthetic Femoral Plate System (Zimmer Biomet, Warsaw, IN, USA) was used for internal fixation in all of these WSP cases. Four out of ten patients had received osteoporosis treatment, and the details of these therapeutic interventions are presented in Table 2.

RBC transfusion was performed in nine of the ten cases (90%), with a mean transfusion volume of 3.8 units. The mean blood loss was 212 mL (50–640 mL), and the mean operative time was 169 min (92–270 min) (Table 2). The estimated blood loss was measured based on intraoperative suction volume and the weight of surgical gauze. The requirement for RBC transfusion may have also been influenced by preoperative anemia, as some patients presented with low hemoglobin levels prior to surgery, in addition to the actual intraoperative blood loss. The average non-weight-bearing period was 4.3 weeks, determined based on bone quality and fixation stability; however, earlier weight bearing was permitted in older patients. Among the nine patients who were able to walk before their surgery, seven regained their ability to walk postoperatively. Although the average hospital stay was 2.8 months, the prolonged duration may be attributed to the institutional setting of Aizu Chuo Hospital, which includes a rehabilitation ward within the same facility that provides subacute care support for various conditions. Regarding postoperative complications, one patient (patient 1) died from aspiration pneumonia 3 months after surgery, and another (patient 7) developed a urinary tract infection during the perioperative period which was successfully treated medically. Of the nine patients who had been living independently at home prior to admission, six were discharged back to their homes. The mean follow-up period was 14.5 months (range: 3–24 months), and no re-fractures were observed in any of the patients after WSP (Table 3).

## 4. Discussion

Peri-implant fractures in the femur are a serious complication that can hinder treatment [8]. In elderly patients, reduced ADLs and prolonged treatment due to non-loading of the lower limb can both lead to disuse syndrome and worsen life expectancy [14]. For elderly patients with bone fragility, the prevention of re-fractures outside the internal fixation material and the possible subsequent ‘fracture cascade’ is therefore key. In cases of BHA/THA periprosthetic fractures, internal fixation is indicated in Vancouver types B1 and C cases because the stem component is stable [15]. However, following internal fixation for periprosthetic hip fractures, 7.4% of Vancouver type B1 hip fractures and 4.0% of type C hip fractures have been reported to occur at different sites on the ipsilateral femur, originating from new trauma [16,17]. Moloney et al. compared a conventional short-plate fixation group (short plate fixation), which did not cover the femoral condyle, with a covered group (spanning plate fixation) for plate fixation of periprosthetic hip fractures of the Vancouver B1 type. They observed neither re-fracture beyond the plate nor plate fracture in the spanning-plate fixation group [16].

It has been reported that a fixation should be strengthened by inserting four bi-cortical screws into each bone fragment or eight or more screws including mono-cortical screws into each bone fragment, or by adding a cable or a locking attachment plate, e.g., by inserting multiple screws into the greater trochanter region as well [18,19,20,21]. Plates should overlap the stem by ≥6 cm in order to reduce the possibility of a fracture outside the implant after internal fixation [22], which can also be applied to Vancouver C type fractures.

In NPPIFs, WSP is indicated as Nail Type 1 or Type 2 [7]. In Type 1 cases with an SFN, revision surgery with a long femoral nail can be considered. However, due to the surgical invasiveness of this technique and concerns about the potential loss of bone stock associated with the removal of the existing intramedullary nail, we opted for plate fixation, even in the cases of Type 1 NPPIFs.

Among periprosthetic femoral TKA fractures, Lewis and Rorabeck classification types 1 and 2 are eligible for internal fixation, for which either a retrograde intramedullary nail or a plate is selected. Retrograde intramedullary nails are particularly good candidates when the fracture involves the diaphysis, but care should be taken to avoid a peri-implant re-fracture caused by stress concentration at the nail tip [23]. As a WSP technique for periprosthetic femoral TKA fractures, it has been suggested that the proximal part of the plate should be long enough to be placed up to the transverse part, and a screw should be inserted into the neck over the plate to prevent future femoral neck fractures [24]; we have adopted this as a WSP method. However, it should be noted that WSP for periprosthetic femoral TKA fractures may be an instance of overtreatment. Our study’s patient 10, who underwent WSP, was an 86-year-old woman with two previous fracture treatments in the contralateral femur and dementia who was able to engage in preoperative ADLs in a wheelchair. Although retrograde intramedullary nailing was technically feasible, this approach would not have addressed the risk of a new peri-implant fracture proximal to the nail. In contrast, WSP allowed for comprehensive reinforcement across the entire femur, including regions at risk of future fractures. Considering her advanced age, comorbidities, and limited functional reserve, WSP was selected to reduce the likelihood of subsequent fractures and associated complications, thereby preventing limb disuse and preserving life expectancy. Compared with other surgical techniques for managing periprosthetic and peri-implant femoral fractures, WSP provides the unique advantage of reinforcing the entire femur, thereby potentially reducing the risk of future ipsilateral fractures beyond the index site. Conventional plate fixation may address the primary fracture but may not offer sufficient structural support to adjacent areas, which remain susceptible to re-fracture due to osteoporosis or implant stress shielding [25]. Retrograde intramedullary nailing is a well-established approach for distal femur fractures but may be limited in patients with poor bone quality, inadequate distal fixation purchase, or preexisting implants obstructing nail insertion [26]. Revision arthroplasty using long-stem prostheses can restore mechanical stability in the setting of prosthetic loosening; however, this technique is associated with an increased risk of postoperative complications, particularly in elderly or medically fragile patients [27]. In contrast, WSP using long locking plates allows for minimally invasive stabilization while spanning areas at high risk of re-fracture, making it a potentially safer alternative in carefully selected high-risk patients. 

Re-fractures in cases of femoral periprosthetic fractures are classified as (i) traumatic periprosthetic femoral re-fractures (T-PFRFs), which occur outside the area of implant placement because of unpredictable trauma associated with reduced bone quality and bone mass, (ii) pathological periprosthetic femoral re-fractures (P-PFRFs), which are a re-fracture of the same fracture site as that of the implant fracture, due to poor fixation and biological activity, infection, and/or bone healing failure [28]. Although the application of WSP in the present patient series was aimed at preventing T-PFRFs, the use of WSP is also expected to improve fixation in cases with a wide peri-stem fracture area and consequently also prevent some P-PFRFs.

According to Lamb et al., the incidence of surgically managed postoperative periprosthetic femoral fractures (POPFF) after THA is 0.92 per 1,000 prosthesis-years, increasing to 1.31 among patients aged 70 years and older [29]. These findings were derived from an extensive analysis of over 800,000 THA cases in the UK National Joint Registry and Hospital Episode Statistics databases [29]. In contrast, within our small cohort of 10 patients treated with WSP for peri-implant femoral fractures, no subsequent ipsilateral femoral fractures were observed over a mean follow-up period of 14.5 months. While the limited sample size and follow-up duration preclude definitive conclusions, this outcome may indicate a potential preventive effect of WSP in high-risk populations, such as elderly individuals with prior implants and compromised bone quality.

We focused on patients aged ≥ 70 years due to the significantly increased risk of fragility fractures in this population. According to recent guidelines, approximately 32.5% of individuals aged ≥ 70 years are at very high fracture risk, based on FRAX estimates [30]. However, we acknowledge that fragility fractures can occur in younger individuals with specific risk factors. For instance, case 6 involved a 54-year-old postmenopausal woman with extensive fracture patterns and compromised bone quality [31], warranting the application of WSP. This suggests that WSP may be beneficial in selected patients under 70 years with high fracture risk. In our study, only four of the ten patients had received pharmacological treatment for osteoporosis. Given the high prevalence of severe osteoporosis in this population, bone health management should be integrated alongside surgical intervention to reduce the risk of subsequent fractures. Postoperative osteoporosis treatment was not standardized in our institution and was initiated on a case-by-case basis at the discretion of the attending orthopedic surgeons and internal medicine specialists, depending on the patient’s general condition, comorbidities, and preoperative management status.

A potential problem with WSP is that it is a highly invasive and complicated surgery that may lead to increased blood loss and prolonged operation times. An average of 3.8 units of concentrated RBC transfusion was required in 90% of the present cases. According to Konda et al., elderly patients undergoing hip fracture surgery required an average transfusion volume of 702 mL, which corresponds to approximately 5.0 units based on the Japanese transfusion standard (1 unit = 140 mL) [32]. This comparison suggests that our observed transfusion volume is relatively modest and within a clinically acceptable range, even in the context of surgically demanding procedures such as WSP. Nonetheless, increased transfusion requirements in elderly trauma patients have been associated with higher rates of complications including cardiac overload, infection, and delayed mobilization [32,33]. Park et al. demonstrated that implementing a Patient Blood Management (PBM) protocol significantly reduced transfusion rates from 52.8% to 36.6% and was associated with improved clinical outcomes in a similar elderly population [33]. In addition to meticulous preoperative planning, it is important to use closed reduction whenever possible and to reduce invasiveness with minimally invasive plate osteosynthesis [34]. Based on these considerations, the results of our analyses indicate that WSP may be useful in preventing postoperative re-fractures in cases with a high risk of re-fracture and in cases in which fixation should be ensured, although efforts to reduce surgical invasiveness are necessary. Furthermore, given that many of the patients in this study were at a high risk of falls, comprehensive fall prevention strategies—including balance training, lower limb strengthening, and home safety assessments—should be incorporated into the postoperative management plan. Such interventions may further reduce the incidence of subsequent fragility fractures and enhance long-term functional recovery.

This study has several limitations. First, the sample size is small, with only 10 patients, which limits the generalizability of our findings. Second, the follow-up period was relatively short, with a mean duration of 14.5 months, which may be insufficient to detect all subsequent fractures or long-term complications. Additionally, the study lacked long-term functional outcome assessments. Patient-reported outcome measures (PROMs) such as the 36-Item Short Form Survey (SF-36) or Western Ontario and McMaster Universities Osteoarthritis Index (WOMAC) were not systematically collected in this preliminary analysis. Future investigations should incorporate these measures to more comprehensively evaluate functional recovery and quality-of-life outcomes in patients undergoing WSP.

## 5. Conclusions

The findings of this study provide meaningful insight into the safety profile and perioperative management of WSP in patients with peri-implant femoral fractures. Although the observed transfusion volume was relatively high, it remained within a clinically acceptable range, and relatively early weight bearing without additional fracture or major complications was achieved in most cases. These results suggest that WSP may be a feasible and safe surgical option when carefully planned and executed, particularly in patients considered to be at high risk of subsequent ipsilateral femoral fracture due to poor bone quality, prior fragility fractures, or extensive implant burden. Although limited by the small sample size and short follow-up period, the present study supports the potential role of WSP in mitigating future fracture risk in this vulnerable patient population.

## Figures and Tables

**Table 1:** The patients’ demographics and fracture characteristics.

Case	Age	Sex	Femoral Implant	Classification	Fracture History	Comorbidities
1	73	M	BHA	Vancouver-Type C	FNF	Chronic renal failure, chronic atrial fibrillation, hypertension
2	71	F	BHA	Vancouver-Type C	FNF, Peripro. FF	Hypertension
3	88	F	THA	Vancouver-Type C	None	Hypertension, severe osteoporosis
4	70	M	THA	Vancouver-Type C	None	Chronic atrial fibrillation, hypertension, valvular heart disease
5	95	F	THA	Vancouver-Type B1	DFF	Hypertension, chronic heart failure, ischemic heart disease, severe osteoporosis
6	54	F	THA	Vancouver-Type B1+C	None	Hypertension
7	93	F	THA + TKA	Vancouver-Type C	None	Cerebrovascular accident, severe osteoporosis
8	91	F	SFN	NPPIF-Type N2A	FTF, PTF, AF	Cerebrovascular accident, severe osteoporosis
9	74	F	SFN	NPPIF-Type N2A	FTF	Arteriovenous malformation
10	86	F	TKA	L&R-Type 2	FNF, FSF	Hypertension, severe osteoporosis

AF; ankle fracture, BHA; bipolar hip arthroplasty, DFF; distal femoral fracture, FNF; femoral neck fracture, FSF; femoral shaft fracture, FTF; femoral trochanteric fracture, L&R; Lewis and Rorabeck, Peripro. FF; periprosthetic femoral fracture, PTF; proximal tibial fracture, SFN; short femoral nail, THA; total hip arthroplasty, TKA; total knee arthroplasty.

**Table 2:** The patients’ perioperative management and surgical data.

Case	ASA-PS	Osteoporosis Treatment History	Medication Details	PRBC (Units)	IOBL (mL)	OT (min)
1	3	No	None	2	220	168
2	2	No	None	4	143	171
3	2	No	None	6	210	138
4	3	Yes	Eldecalcitol	2	640	209
5	2	No	None	2	50	92
6	2	No	None	8	172	252
7	3	Yes	Teriparatide	4	289	146
8	2	Yes	Eldecalcitol, calcium aspartate, denosumab	0	99	113
9	3	Yes	Alendronate	4	130	270
10	2	No	None	6	170	132

ASA-PS; American Society of Anesthesiologists physical status, IOBL; intraoperative blood loss, OT; operative time, PRBC; packed red blood cell transfusion.

**Table 3:** The patients’ postoperative course and outcomes.

Case	NWBP (Week)	Pre-Injury ADLs	Final ADLs	LHSD (Month)	Complications	Pre-ALS	Discharge Destination	FUP (Month)	Periprosthetic Re-Fracture
1	4	Independent Walking	Wheelchair	3	AP, Death	Home	Died at home	3	None
2	8	Independent Walking	Independent Walking	3	None	Home	Home	21	None
3	4	Walker	Wheelchair	4	None	Home	Nursing home	24	None
4	4	Independent Walking	Independent Walking	2	None	Home	Home	12	None
5	2	Walker	Walker	2	None	Home	Home	13	None
6	8	Independent Walking	Independent Walking	3	None	Home	Home	18	None
7	2	Walker	Walker	3	UTI	Home	Nursing home	12	None
8	1	Cane	Assisted Walking	2	None	Home	Home	12	None
9	4	Cane	Walker	3	None	Home	Home	18	None
10	6	Wheelchair	Wheelchair	2.5	None	Nursing home	Nursing home	12	None

ADLs; activities of daily living, AP; aspiration pneumonia, LHSD; length of hospital stay, NWBP; non-weight-bearing period, UTI; urinary tract infection, FUP; follow-up period, Pre-ALS; pre-admission living situation.

## Data Availability

The original contributions presented in this study are included in the article. Further inquiries can be directed to the corresponding author.

## References

[1] Nakatani H. (2023). Ageing and shrinking population: The looming demographic challenges of super-aged and super-low fertility society starting from Asia. Glob. Health Med..

[2] Ackerman I.N., Bohensky M.A., Zomer E., Tacey M., Gorelik A., Brand C.A., de Steiger R. (2019). The projected burden of primary total knee and hip replacement for osteoarthritis in Australia to the year 2030. BMC Musculoskelet. Disord..

[3] Coughlan T., Dockery F. (2014). Osteoporosis and fracture risk in older people. Clin. Med..

[4] Patsiogiannis N., Kanakaris N.K., Giannoudis P.V. (2021). Periprosthetic hip fractures: An update into their management and clinical outcomes. EFORT Open Rev..

[5] Canton G., Ratti C., Fattori R., Hoxhaj B., Murena L. (2017). Periprosthetic knee fractures. A review of epidemiology, risk factors, diagnosis, management and outcome. Acta Biomed..

[6] Hemmann P., Friederich M., Körner D., Klopfer T., Bahrs C. (2021). Changing epidemiology of lower extremity fractures in adults over a 15-year period—A National Hospital Discharge Registry study. BMC Musculoskelet. Disord..

[7] Chan L.W.M., Gardner A.W., Wong M.K., Chua K., Kwek E.B.K., Singapore Orthopaedic Research CollaborativE (SORCE) (2018). Non-prosthetic peri-implant fractures: Classification, management and outcomes. Arch. Orthop. Trauma Surg..

[8] Randelli F., Pace F., Priano D., Giai Via A., Randelli P. (2018). Re-fractures after periprosthetic femoral fracture: A difficult to treat growing evidence. Injury.

[9] Lane J.M., Witayakom W. (2023). What’s new in osteoporosis and fragility fractures. J. Bone Jt. Surg. Am..

[10] Duncan C.P., Masri B.A. (1995). Fractures of the femur after hip replacement. Instr. Course Lect..

[11] Rorabeck C.H., Taylor J.W. (1999). Classification of periprosthetic fractures complicating total knee arthroplasty. Orthop. Clin. N. Am..

[12] Takai H., Nonoue Y., Kitajima M., Hama S., Takahashi T. (2022). Total femur fixation using the ‘nail-plate docking technique’ for ipsilateral femur shaft fracture. Trauma Case Rep..

[13] Kenny P., Rice J., Quinlan W. (1998). Interprosthetic fracture of the femoral shaft. J. Arthroplast..

[14] Aarden J.J., van der Esch M., Engelbert R.H.H., van der Schaaf M., de Rooij S.E., Buurman B.M. (2017). Hip fractures in older patients: Trajectories of disability after surgery. J. Nutr. Health Aging.

[15] Chakravarthy J., Bansal R., Cooper J. (2007). Locking plate osteosynthesis for Vancouver Type B1 and Type C periprosthetic fractures of femur: A report on 12 patients. Injury.

[16] Moloney G.B., Westrick E.R., Siska P.A., Tarkin I.S. (2014). Treatment of periprosthetic femur fractures around a well-fixed hip arthroplasty implant: Span the whole bone. Arch. Orthop. Trauma Surg..

[17] Füchtmeier B., Galler M., Müller F. (2015). Mid-term results of 121 periprosthetic femoral fractures: Increased failure and mortality within but not after one postoperative year. J. Arthroplast..

[18] Corten K., Vanrykel F., Bellemans J., Frederix P.R., Simon J.P., Broos P.L. (2009). An algorithm for the surgical treatment of periprosthetic fractures of the femur around a well-fixed femoral component. J. Bone Jt. Surg..

[19] Stoffel K., Sommer C., Meyer C., Schnettler R., Schütz M., Perka C., Ruedi T.P. (2013). Chapter5.4 Internal fixation. Periprosthetic Fracture Management.

[20] Lenz M., Perren S.M., Gueorguiev B., Richards R.G., Hofmann G.O., Fernandez dell’Oca A., Höntzsch D., Windolf M. (2014). A biomechanical study on proximal plate fixation techniques in periprosthetic femur fractures. Injury.

[21] Kim M.B., Cho J.W., Lee Y.H., Shon W.Y., Park J.W., Oh J.K. (2017). Locking attachment plate fixation around a well-fixed stem in periprosthetic femoral shaft fractures. Arch. Orthop. Trauma Surg..

[22] Walcher M.G., Giesinger K., du Sart R., Day R.R., Kuster M.S. (2016). Plate positioning in periprosthetic or interprosthetic femur fractures with stable implants—A biomechanical study. J. Arthroplast..

[23] Hou Z., Bowen T.R., Irgit K., Strohecker K., Matzko M.E., Widmaier J., Smith W.R. (2012). Locked plating of periprosthetic femur fractures above total knee arthroplasty. J. Orthop. Trauma.

[24] Shon O.J., Cho S.J., Kim G.B. (2023). Long locking plate combined with locking attachment plate in patients with periprosthetic femoral fracture around ipsilateral stem after total knee arthroplasty. BMC Musculoskelet. Disord..

[25] Kösters C., den Toom D., Metzlaff S., Daniilidis K., Barz L., Roßlenbroich S. (2022). Peri- and Interprosthetic Femoral Fractures—Current Concepts and New Developments for Internal Fixation. J. Clin. Med..

[26] Kim J., Kang S.B., Nam K., Rhee S.H., Won J.W., Han H.S. (2012). Retrograde intramedullary nailing for distal femur fracture with osteoporosis. J. Orthop. Case Rep..

[27] Sponer P., Korbel M., Grinac M., Prokes L., Bezrouk A., Kucera T. (2021). The outcomes of cemented femoral revisions for periprosthetic femoral fractures in elderly: Comparison with cementless stems. Clin. Interv. Aging.

[28] Vicenti G., Bizzoca D., Solarino G., Carrozzo M., Belluati A., D’Arienzo A., De Carolis O., Moretti B. (2023). Periprosthetic femoral re-fractures pathogenesis, classification, and surgical implications. Injury.

[29] Lamb J.N., Evans J.T., Relton S., Whitehouse M.R., Wilkinson J.M., Pandit H. (2024). The incidence of postoperative periprosthetic femoral fracture following total hip replacement: An analysis of UK National Joint Registry and Hospital Episodes statistics data. PLoS Med..

[30] Gregson C.L., Compston J.E. (2022). New national osteoporosis guidance-implications for geriatricians. Aging Clin. Exp. Res..

[31] Charde S.H., Joshi A., Raut J. (2023). A comprehensive review on postmenopausal osteoporosis in women. Cureus.

[32] Konda S.R., Perskin C.R., Parola R., Robitsek R.J., Ganta A., Egol K.A. (2021). Trauma risk score also predicts blood transfusion requirements in geriatric hip fracture patients. Geriatr. Orthop. Surg. Rehabil..

[33] Kim J.H., Shin H.J., You H.S., Park Y., Ahn K.H., Jung J.S., Han S.B., Park J.H., Korea University Bloodless Medicine Center Scientific Committee (2023). Effect of a patient blood management program on the appropriateness of red blood cell transfusion and clinical outcomes in elderly patients undergoing hip fracture surgery. J. Korean Med. Sci..

[34] Min B.W., Cho C.H., Son E.S., Lee K.J., Lee S.W., Min K.K. (2018). Minimally invasive plate osteosynthesis with locking compression plate in patients with Vancouver type B1 periprosthetic femoral fractures. Injury.

